# CHF6467 Exerts Neuroprotective Effects in the Rat Model of Acute Ischemic Stroke

**DOI:** 10.3390/brainsci16080839

**Published:** 2026-08-07

**Authors:** Chiara Demartini, Fabrizio Facchinetti, Fabio Blandini, Cristina Tassorelli, Francesca Malerba, Antonino Cattaneo, Diana Amantea, Bruno P. Imbimbo, Rosaria Greco

**Affiliations:** 1 Section of Translational Neurovascular Research, IRCCS Mondino Foundation, Via Mondino, 2, 27100 Pavia, Italy; chiara.demartini@mondino.it (C.D.); cristina.tassorelli@unipv.it (C.T.); 2 Research & Development, Chiesi Farmaceutici, Largo Belloli 11/A, 43122 Parma, Italy; f.facchinetti@chiesi.com (F.F.); b.imbimbo@chiesi.com (B.P.I.); 3 Department of Brain and Behavioral Sciences, University of Pavia, Via Bassi 21, 27100 Pavia, Italy; fabio.blandini@policlinico.mi.it; 4 Ca’Granda IRCCS Foundation-Ospedale Maggiore Policlinico, Via Francesco Sforza 28, 20122 Milan, Italy; 5 Fondazione EBRI—Rita Levi-Montalcini, Viale Regina Elena, 295, 00161 Rome, Italy; f.malerba@ebri.it (F.M.); a.cattaneo@ebri.it (A.C.); 6 Institute of Nanotechnology National Research Council (CNR Nanotec) c/o Campus Ecotekne, Via Monteroni, 73100 Lecce, Italy; 7 Section of Preclinical and Translational Pharmacology, Department of Pharmacy, Health and Nutritional Sciences, University of Calabria, Via Savinio, I-87036 Rende, CS, Italy; diana.amantea@unical.it

**Keywords:** brain ischemia, CHF6467, tMCAo, rat

## Abstract

**Highlights:**

**What are the main findings?**

- CHF6467: reduced infarct volume and improved neurological function following focal cerebral ischemia.
- CHF6467-treated rats showed a higher probability of achieving a favorable neurological outcome seven days after permanent cerebral ischemia.

**What are the implications of the main findings?**

- The observed reduction in infarct volume was directly accompanied by improved functional neurological recovery.
- CHF6467 represents a promising intranasal neuroprotective candidate for acute ischemic stroke therapy.
- Further studies are warranted to fully characterize its regional brain distribution and elucidate its precise molecular mechanisms of action.

**Abstract:**

**Objectives:** The critical role of nerve growth factor (NGF) in neuroprotection has been demonstrated in preclinical models of neuronal injury. Here, we investigated the neuroprotective effects of intranasal CHF6467, a recombinant mutant form of human NGF lacking algogenic activity, in transient (45 min) middle cerebral artery occlusion (tMCAo). **Methods**: Male Wistar rats (*n* = 20 per group) were treated intranasally with CHF6467 (20 µg/kg, 40 µL) or vehicle (0.9% saline, 40 μL). The first dose was administered 15 min after the onset of MCAo and the second dose 24 h later. Neurological behavioral tests were performed 24 and 48 h after tMCAo. Infarct volume was measured 48 h after tMCAo. Additionally, a separate cohort of rats underwent permanent MCAo (pMCAo) and received two intranasal doses of CHF6467 or vehicle, with long-term neurological outcome assessed 7 days later using the De Simoni neuroscore (*n* = 13–14 per group). CHF6467 levels were measured in intact brain samples from CHF6467-treated rats (*n* = 5) and vehicle-treated controls (*n* = 4). Samples were collected 2 h after the last administration. **Results:** CHF6467 significantly improved neurological and sensorimotor performance compared with the vehicle-treated rats. This functional effect was associated with a reduction in infarct volume that was positively correlated with neurological deficit improvement. In the pMCAo cohort, CHF6467-treated rats were more likely to achieve a favorable neurological outcome at seven days compared with the vehicle-treated animals. Intranasal CHF6467 reached the brain but showed variable and non-uniform distribution across regions. Levels were highest in the olfactory bulbs, while the cortex and striatum had lower concentrations. In vehicle-treated rats, the drug was undetectable. **Conclusions**: This study demonstrates the neuroprotective effects of CHF6467 against ischemic brain injury, evidenced by both functional improvement and reduction in infarct volume. Although it reaches the brain, its distribution is heterogeneous and assessed only at one time-point. Further studies should examine its longer-term distribution and underlying mechanisms.

## 1. Introduction

Cerebral ischemia is the second leading cause of death, and although mortality is decreasing in Western countries, the increasing incidence of stroke associated with population aging is one of the most serious health and social problems. Effective drugs for the treatment of acute ischemic stroke remain limited. Only a small percentage of stroke patients can benefit from revascularization procedures due to the numerous complications and the short time window for intervention, highlighting the urgent need to develop new treatments to stop the progression of irreversible damage in the ischemic penumbra [1].

Ischemic stroke triggers angiogenesis and vascular remodeling plays a role in the complex process of brain regeneration after ischemic damage, including increasing synaptic plasticity and restoring cerebral blood flow. According to clinical data, patients with cerebral infarction have a large increase in microvessel numbers in their penumbra, and higher microvessel density may be associated with longer post-stroke survival times [2].

Nerve growth factor (NGF) is a neuroprotective neuropeptide with well-established neurotrophic and anti-apoptotic effects on cholinergic neurons [3,4]. Although NGF was initially discovered as a neurotrophic factor, increasing evidence has shown that it may also affect both physiological and pathologic processes of angiogenesis [2,5]. NGF is produced by astrocytes and microglia, and its expression is markedly upregulated by local tissue damage [6] to modulate inflammation. It can induce anti-inflammatory effects by reducing pro-inflammatory cytokine release while increasing levels of anti-inflammatory soluble mediators (e.g., interleukin-10) [7]. The high molecular weight of NGF severely limits its ability to cross the blood–brain barrier (BBB), representing a major obstacle to its clinical use for the treatment of neurological disorders. A valid alternative approach that bypasses the BBB consists of intranasal administration; indeed, recent studies have shown that a significant amount of NGF can reach the central nervous system via this route [8,9,10]. Intranasal administration offers several benefits over traditional procedures. The olfactory route represents an advantageous option as it is readily applicable, safe, and minimizes systemic exposure and associated adverse effects.

Intranasal NGF has been tested in models of cerebral ischemia, whereby it was reported to induce neurogenesis in the ischemic core without reducing the infarct volume at late time points [11]. By contrast, a reduction in infarct volume through anti-apoptotic mechanisms was reported in another study [12]. NGF induces neuroprotection after oxygen-glucose deprivation in an in vitro model of cerebral ischemia, through upregulation of heme oxygenases-1, which is known to promote neuronal survival [13].

Despite its neuroprotective effects, NGF displays a marked algogenic activity [14,15]. Indeed, the use of wild-type NGF in the clinical setting was discontinued after reports of potent dose-dependent hyperalgesia, including back pain and severe myalgia [16,17]. To make the most of NGF’s neuroprotective potential, avoiding the risk of hyperalgesia-related side effects, a painless recombinant mutant form of human NGF, called CHF6467, has recently been developed. CHF6467 has neurotrophic and neuroprotective properties identical to wild-type NGF but with at least ten times less algogenic activity [18,19].

Here, in an animal model of cerebral ischemia, we tested the hypothesis that CHF6467 may have the same therapeutic potential as wild-type NGF in acute stroke.

## 2. Materials and Methods

### 2.1. Animals

All animal experiments were performed and reported following the Animal Research: Reporting of in vivo Experiments (ARRIVE) guidelines [20]. Animal care and experimental procedures were carried out following the guidelines of the Italian Ministry of Health (DL 26/2014) and the European Directive 2010/63/UE. The animals were housed in the animal facility of the University of Pavia (Pavia, Italy) and of the University of Calabria in groups of two per cage under controlled conditions (i.e., temperature 21–22 °C, 50–60% relative humidity) and a 12/12 h light cycle (with lights on at 7.00 am). Food and water were provided ad libitum. Upon arrival, the animals were acclimated to the housing conditions for one week prior to the experimental tests. To help reduce stress, elements from the home nest, such as nesting paper and cardboard rolls, were placed in the recovery box. Experiments and analysis were performed in a randomized fashion by an experimenter blinded to treatment.

All the experiments were performed on male Wistar rats (7–10 weeks old, corresponding to the body weight range of 260–340 g, Charles River, Calco, Lecco, Italy. The rats were housed in sterile cages and allowed free movement and access to food and water ad libitum throughout the experiment. The animal humane endpoint was defined for animals that exceeded 25% loss of baseline weight. The protocol was approved by the Committee set by the Ministry of Health at the National Institute of Health (Authorizations No. 98/2018-PR of 12 February 2018 and No. 214/2025-PR of 20 March 2025).

### 2.2. Experimental Design

**Figure 1:**
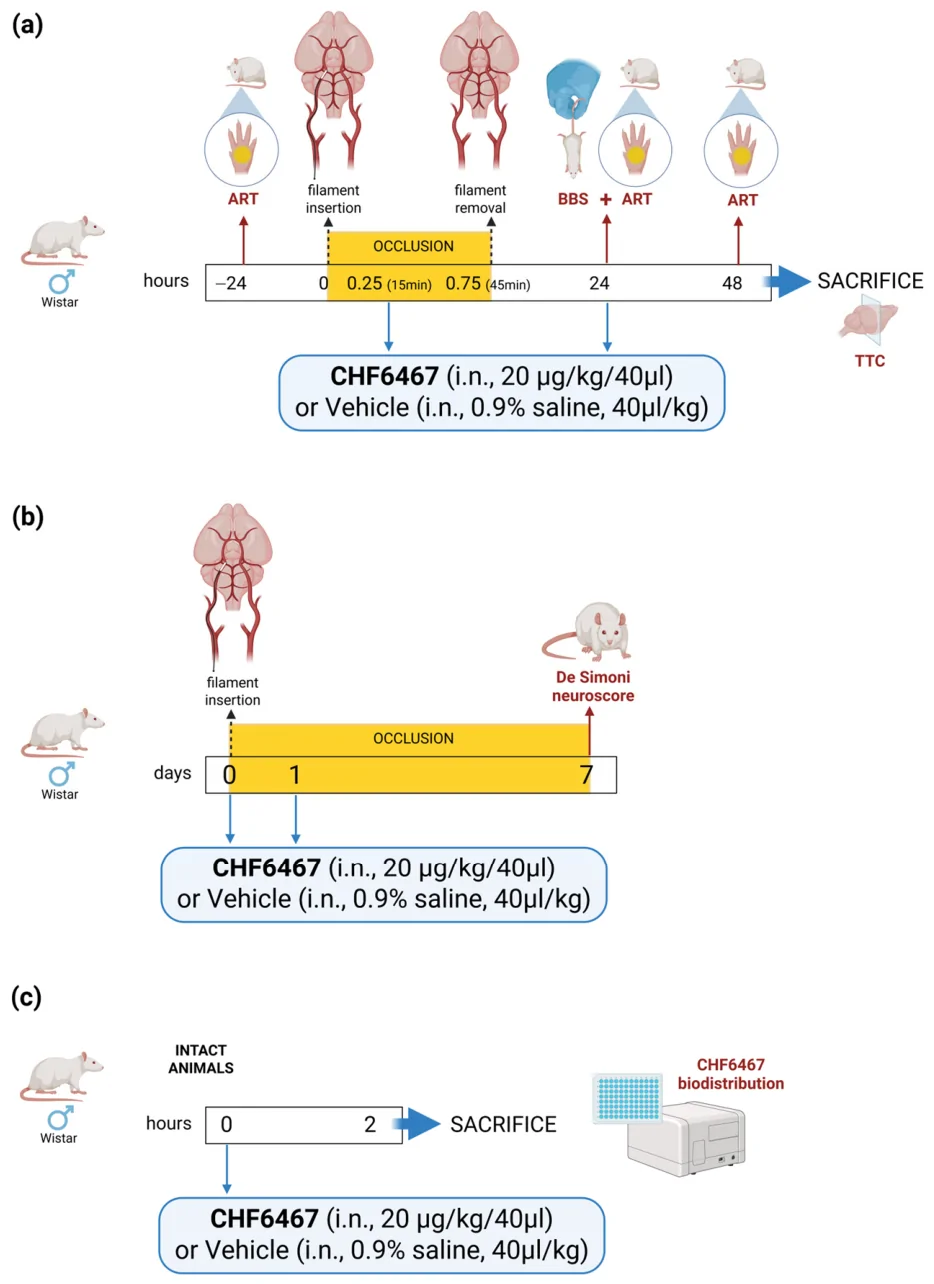
Experimental design. (**a**) tMCAO cohort: the day before tMCAo rats were tested with the adhesive removal test (ART) for basal values. The first administration of CHF6467 or vehicle was performed 15 min after induction of tMCAo (filament insertion). After 45 min of occlusion, the filament was removed to allow reperfusion. Twenty-four hours after tMCAo, animals were tested for baseline behavioral score (BBS) and ART, and then the second intranasal administration of CHF6467 or vehicle was performed. Forty-eight hours after tMCAo, animals were tested with ART and then were sacrificed for sample collection and processing for 2,3,5-triphenyltetrazolium chloride (TTC) vital staining. (**b**) pMCAO cohort: the two administrations of CHF6467 or vehicle were performed 15 min and 24 h after induction of pMCAo (filament insertion). Seven days after pMCAo, animals were tested for the De Simoni neuroscore. (**c**) Biodistribution cohort: intact rats were treated with a single intranasal administration of CHF6467 or vehicle and then sacrificed two hours later for sample collection and processing for CHF6467 biodistribution analysis.

To assess the neuroprotective effects of CHF6467 (provided by Chiesi Farmaceutici) in acute stroke, rats were subjected to either transient (45 min) middle cerebral artery occlusion (tMCAo) or permanent (7-day) MCAo (pMCAo) and received two intranasal treatments. Specifically, rats were treated with CHF6467 (20 µg/kg/40 µL) or vehicle (0.9% saline, 40 µL/kg) 15 min after MCAo onset and then 24 h later [12,21] (Figure 1). The second administration (at 24 h) was performed after behavioral testing.

The overall experimental workflow comprised three distinct cohorts:

1. tMCAo cohort (*n* = 20 per group): To evaluate acute neurological deficits and the potential neuroprotective effects of CHF6467, rats underwent behavioral evaluation using the adhesive removal test and a neurological score. The adhesive removal test was conducted the day before surgery (baseline) and again at 24 h and 48 h after tMCAo. The neurological score was recorded at 24 h post-tMCAo. At the conclusion of the final behavioral assessment (48 h post-surgery), animals were sacrificed, and their brains were collected for infarct volume quantification (primary outcome) via 2,3,5-triphenyltetrazolium chloride (TTC) staining (Figure 1a).

2. pMCAo cohort (*n* = 16 per group): A separate pMCAo cohort was established to evaluate the long-term functional effects of CHF6467 over 7 days, assessing neurological recovery using the De Simoni neuroscore (Figure 1b).

3. Biodistribution cohort: To quantify drug penetration in the brain, a separate biodistribution sub-study was conducted using intact, non-ischemic rats treated with either intranasal CHF6467 (*n* = 5) or a vehicle (*n* = 4) for regional brain drug-level analysis two hours after administration (Figure 1c).

The investigators responsible for volume measurement and behavioral assessment were unaware of the treatment assignment. Exclusion criteria were prespecified. These were (1) development of subarachnoid hemorrhage or hemorrhagic transformation after treatment determined at the moment of sacrifice, and (2) major violations of the experimental protocol: surgical errors or complications (e.g., major arterial or venous hemorrhage, vagus nerve section, carotid artery dissection, entrapment or filament displacement) during the MCAo procedure; errors in the timing of ischemia. Body weight was monitored throughout the experimental period as an indicator of systemic toxicity. In addition, body weight was assessed in conjunction with a thorough observation of general clinical conditions, including general appearance, posture, spontaneous activity, food and water intake, hydration status, and neurological condition.

### 2.3. Focal Cerebral Ischemia Model

MCAo was achieved using the intraluminal filament technique [22]. Briefly, for tMCAo, the right middle cerebral artery was occluded for 45 min before withdrawal of the thread to allow reperfusion for the remainder of the experiment (48 h) (Figure 1a). For the assessment of long-term neuroprotection, rats were subjected to 7 days of pMCAo (Figure 1b). Briefly, rats were anesthetized (for tMCAo: tiletamine, 100 mg/kg, i.m., xylazine 4 mg/kg i.p.; for pMCAO: 3% isoflurane vaporized in air) and their body temperature monitored with a rectal probe maintained at 37 °C throughout the surgical operation with a heating pad. A neck incision was made and, under an operating microscope, the external and internal right carotid arteries were isolated. A silicone-coated nylon filament (diameter: 0.37 mm, Doccol Corporation, Redlands, CA, USA) was introduced through the external carotid artery and advanced approximately 19 mm from the carotid bifurcation to occlude the origin of the right MCA. To allow reperfusion, rats were re-anesthetized for filament removal and then were kept in their cages with free access to food and water.

### 2.4. Behavioral Tests

#### 2.4.1. Basic Behavioral Scale

Bederson scores were used to evaluate neurological function in all rats 24 h after tMCAo (Figure 1a), as previously described [23]. The basic behavioral scale assignment ranged from 0 to 4, in which 0: no observable deficit; 1: forelimb flexion; 2: decreased resistance to lateral push and forelimb flexion; 3: same behavior as 2 with circling; 4: cannot walk spontaneously or seizure activity.

#### 2.4.2. Adhesive Removal Test

The adhesive removal test has been used as a measure of motor coordination and sensory neglect after stroke in experimental studies [24,25]. Since it was previously reported that most animals are unable to perform the test on the first day of testing, we evaluated the adhesive removal test on the second day (48 h) as well (Figure 1a). All animals were acclimatized to the test procedure in the week before surgery via 3 training sessions of 3 tests. Each rat was lightly restrained to allow the attachment of a 10 mm circular office sticker to the palm of each forepaw. The order of attachment (left or right applied first) was alternated across tests. Immediately after the placement of both stickers, a gentle pressure was simultaneously applied to both forelimbs to minimize bias. Then the animal was placed in the observation box and their behavior was recorded with a video camera for offline analysis. Three trials (of 3 min duration each) were undertaken in each test session, with a rest period of at least 1 min between trials. The time to contact and remove each sticker was recorded. If the stickers could not be removed within this time window, a maximum time (180 s) was assigned.

#### 2.4.3. De Simoni Neuroscore

Neurological function was assessed after 7 days of pMCAo using the De Simoni neuroscore (Figure 1b), a standardized composite test designed specifically to evaluate sensorimotor deficits following experimental stroke in rodents. The score assesses several domains of neurological function, including spontaneous activity, limb symmetry, gait, balance, coordination, and sensorimotor responses [26,27]. Individual test scores are summed to generate a composite neurological score that ranges from 0 (no detectable deficit) to 56 (the worst performance across all categories).

### 2.5. Quantification of Ischemic Damage

Cerebral infarct volume was evaluated 48 h after reperfusion in all rats subjected to 45 min MCAo (treated with vehicle or CHF6467), using the 2,3,5-triphenyltetrazolium chloride (TTC) vital staining method. Rats were sacrificed and their brains were rapidly dissected to obtain eight serial coronal sections, cut at 2 mm intervals from the frontal pole, using a rat brain matrix (RBMA-300C, 2 Biological Instruments, Besozzo, Italy). Brain slices were stained in a solution containing 2% TTC in saline, at 37 °C for 10 min, and then fixed in 4% neutral-buffered formaldehyde at 4 °C. Images of TTC-stained sections were captured using a digital scanner and analyzed using image analysis software (ImageJ, version 1.30). The infarct volume (mm3) was calculated by summing the infarcted area (unstained) of the eight sections and multiplying the obtained value by the interval thickness between sections [22].

### 2.6. AlphaLISA Quantification of CHF6467

An AlphaLISA immunoassay was set up to selectively detect CHF6467 in rat brain homogenates in the presence of endogenous rat NGF.

Brain tissues (olfactory bulbs, cortex, striatum, hippocampus) were collected from nine experimental rats, of which five were treated with CHF6467 and four with vehicle.

In addition, one naïve rat was included exclusively for assay setup and validation. The naïve brain was homogenized as a whole and used to assess matrix effects, background signal, and spike recovery performance.

Rats received intranasal CHF6467/vehicle once daily for two consecutive days and were sacrificed 2 h after the last administration. Brain tissues were homogenized in Tris-based lysis buffer (2.5 mL/g tissue; 0.1 M Tris-HCl, 0.4 M NaCl, 0.1% SDS, 1% Triton X-100 and protease inhibitors Roche), incubated on ice for 30 min, centrifuged (15,000× *g*, 60 min, 4 °C), and total protein concentration was determined by Bradford assay.

The assay exploited the mutation-specific monoclonal antibody 4GA (biotinylated) and αD11 (conjugated to AlphaLISA acceptor beads). Antibody biotinylation and conjugation to acceptor beads were carried out following the manufacturer’s instructions. Calibration curves were generated using recombinant CHF6467 (purified as previously described [19]) diluted in lysis buffer (dynamic range 0.5 ng/mL–13 pg/mL). For each well, 5 µL of sample or standard was incubated with 5 µL of AlphaLISA immunoassay buffer containing biotinylated 4GA (3 nM final concentration) and αD11-conjugated acceptor beads (10 µg/mL final concentration). Plates were incubated overnight at 4 °C. Donor beads were then added to reach a final concentration of 50 µg/mL, followed by 30 min incubation at room temperature in the dark. Signal was acquired using an EnSpire Alpha plate reader.

To validate assay performance in brain matrix, spike-recovery experiments were performed by adding recombinant CHF6467 (0.1 ng/mL final concentration) to pooled rat brain homogenates. Samples were assayed neat and at ≥2 dilutions in duplicate. Recovery (%) was calculated by comparing measured versus expected concentrations. Signal-to-noise ratio (S/N) and limit of quantification were determined for each calibration curve.

### 2.7. Statistics and Analysis

The study’s sample size was calculated based on the hypothesized effect of CHF6467 on brain infarct volume. A mean infarct volume of 350 mm^3^ was assumed for the vehicle group, whereas CHF6467 was expected to reduce this value to 240 mm^3^. The common standard deviation for both groups was assumed to be 103 mm^3^. Assuming a type I error of 5% and a power of 80%, at least 16 animals per group. To account for potential missing data of up to 25%, 20 animals were included in each group. In this cohort, 8 rats were excluded due to training failure in the adhesive removal test, leaving *n* = 16 per group for functional analysis. For the long-term pMCAo cohort, peri-operative mortality (*n* = 3 vehicle, *n* = 2 CHF6467) resulted in a final sample size of *n* = 13 vehicle-treated and *n* = 14 CHF6467-treated rats for 7-day De Simoni neuroscore evaluation.

Statistical analyses were performed using GraphPad Prism (version 9.3.1). Data normality was evaluated with the Shapiro–Wilk test. Non-normally distributed variables (infarct volume, Bederson score, and De Simoni neuroscore) were analyzed using the Mann–Whitney rank sum test. Sensorimotor performance in the adhesive removal test was analyzed via two-way repeated-measures ANOVA (fixed factors: “time”, “treatment”, and their interaction). Differences in the proportion of animals achieving favorable neurological recovery (De Simoni score ≤ 27) were evaluated using two-sided Fisher’s exact test, with relative risk (RR) and 95% confidence intervals (CI) reported. A *p* ≤ 0.05 was considered statistically significant.

## 3. Results

### 3.1. Neurological Deficit Analysis

**Figure 2:**
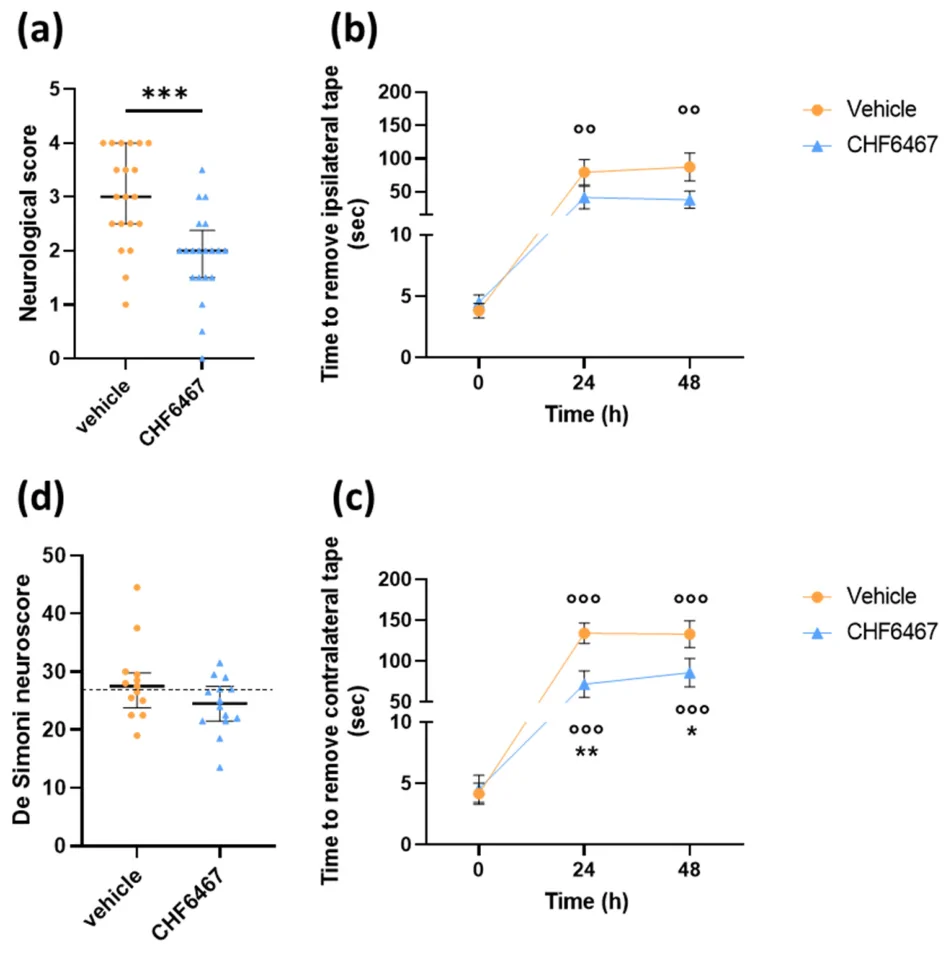
Neurological deficit analysis. (**a**) Basic behavioral scale 24 h after tMCAo. Data are expressed as median with interquartile range. Mann–Whitney rank sum test; *** *p* = 0.0003 CHF6467 vs. Vehicle; *n* = 20. (**b**) Adhesive removal test in ipsilateral (right) forepaw at baseline (0 h), 24 h and 48 h after tMCAo. °° *p* < 0.01 vs. baseline (0 h) of the vehicle group; (**c**) adhesive removal test in contralateral (left) forepaw at baseline (0 h), 24 h and 48 h after tMCAo. °°° *p* < 0.001 24 h vs. baseline (0 h) of both the vehicle and CHF6467 groups; * *p* < 0.05 and ** *p* < 0.01 CHF6467 vs. Vehicle. For B and C, data are expressed as mean ± SEM. Two-way analysis of variance for repeated measures [with fixed factors for “time” (Baseline, 24 h, 48 h), “treatment” (CHF6467 and Vehicle), and their interaction] followed by Šídák’s multiple comparisons test; *n* = 16. (**d**) De Simoni neuroscore (expressed as median with interquartile range) assessed after 7 days of MCAo. Mann–Whitney rank sum test; Dotted line indicates the threshold for severe neuroscore (i.e., >27); *n* = 13–14.

Rats treated with vehicle showed a significant neurological deficit, evaluated by Bederson scores, 24 h after tMCAo compared with the CHF6467 group (Figure 2a). In the adhesive removal test, prior to surgery, all animals were able to remove the tape rapidly and did not show preference in the order in which tapes were removed. Following tMCAo, the time required to remove the tape increased in both groups, 24 h and 48 h post-surgery, contralaterally (Figure 2c) and, to a lesser extent, ipsilaterally to the lesioned hemisphere, with significance only for the vehicle group (Figure 2b). However, such an increase was significantly less pronounced in the animals that received CHF6467, both at 24 h and 48 h post-tMCAo, with a significant difference compared with the vehicle group at the contralateral side (Figure 2c). Neurological outcome was assessed seven days after pMCAO using the De Simoni neurological score (Figure 2d). Following two intranasal administrations of CHF6467 (15 min and 24 h after ischemia induction), 78.6% of CHF6467-treated animals achieved a favorable neurological outcome (predefined as a total neurological score ≤ 27), compared with 46.2% of vehicle-treated animals. Thus, rats treated with CHF6467 were 1.70-fold more likely to exhibit functional recovery than those receiving vehicle (estimated relative risk, RR = 1.70, 95% CI 0.89–3.25; two-sided Fisher’s exact test, *p* = 0.120).

### 3.2. Infarct Volume Analysis

**Figure 3:**
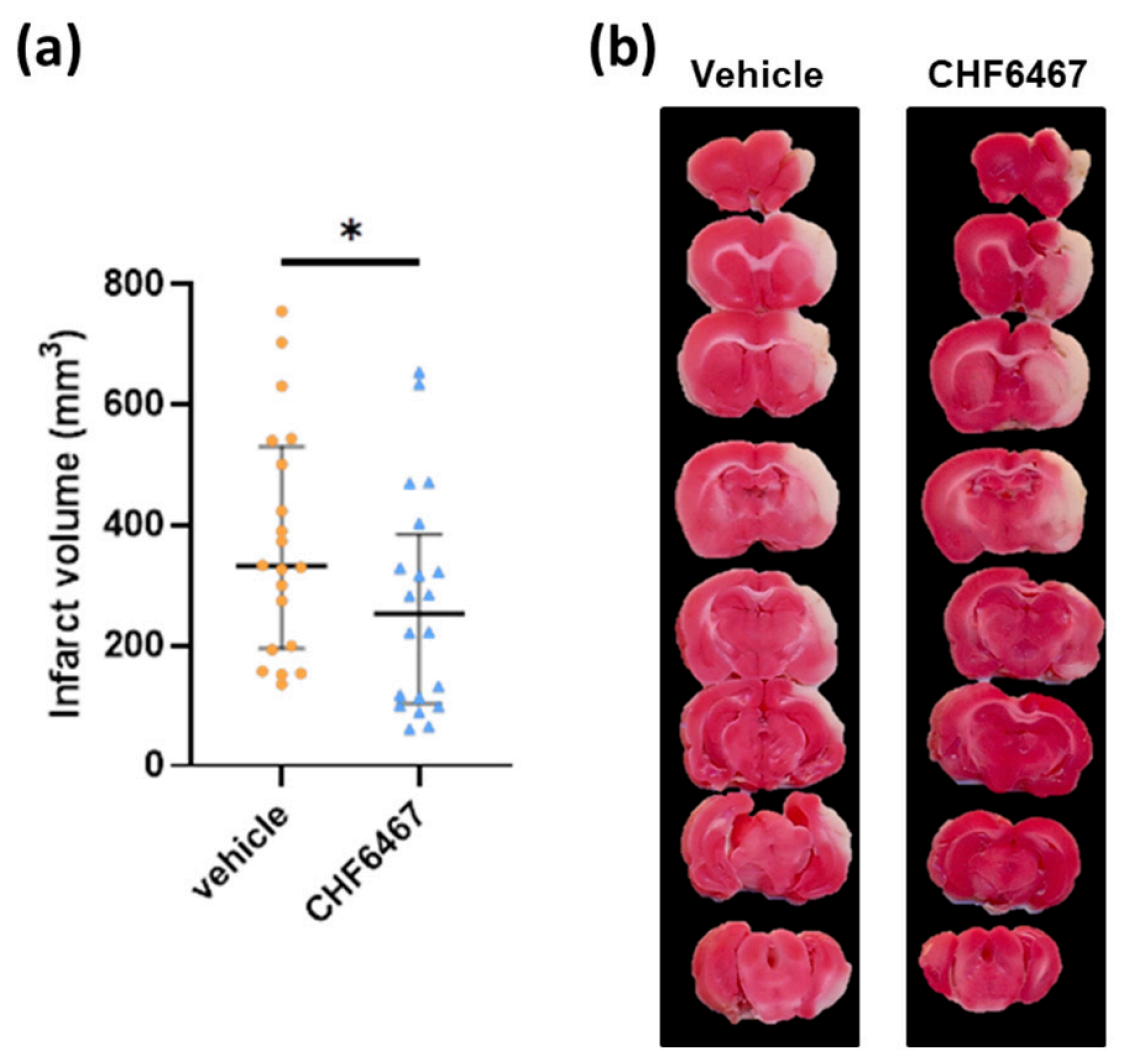
Analysis of the infarct volume at 48 h after the tMCAo. (**a**) Brain ischemic volume expressed in mm^3^. (**b**) Representative coronal brain slices stained with 2,3,5-triphenyltetrazolium chloride (TTC) of vehicle- and CHF6467-treated animals. Viable, metabolically active tissue is stained deep red due to production of formazan by mitochondrial dehydrogenase activity, whereas ischemic/infarcted tissue remains unstained (pale/white). Consequently, larger pale areas reflect more extensive cerebral ischemia/reperfusion damage. Data are expressed as median with interquartile range. Mann–Whitney rank sum test. * *p* = 0.05, *n* = 20.

Treatment with intranasal CHF6467 induced a median reduction in infarct volume of approximately 24.0% with respect to the vehicle group 48 h after tMCAo (vehicle: 332.1 [194.8–529.8] vs. CHF6467: 252.3 [103.3–384.1]; *p* = 0.05, Mann–Whitney test), with a moderate effect size (rank-biserial correlation, r_rβ_= 0.36). Data are reported in Figure 3.

### 3.3. CHF6467 Brain Distribution

The AlphaLISA assay showed an adequate dynamic range (13–500 pg/mL), acceptable S/N ratios, and reliable spike-recovery performance in rat brain homogenates, confirming matrix compatibility. Recovery values were within acceptable limits, supporting assay accuracy in complex tissue lysates.

With the exception of one rat, intranasal CHF6467 reached the brain, but its distribution across regions was not homogeneous. We observed inter-animal variability, with detectable levels appearing in different areas, such as the cortex in some rats and striatum in others, while the highest and most consistent concentrations were detected in the olfactory bulbs. Most other regions, including all hippocampal samples, remained below the detection limit (Table 1). Importantly, all samples were analyzed in a blinded manner, and treatment allocation was disclosed only after completion of the measurements. Upon unblinding, all samples showing detectable CHF6467 levels corresponded to treated animals, whereas vehicle-treated animals were consistently negative, supporting the assay specificity.

## 4. Discussion

After stroke, reparative and regenerative drugs that enhance endogenous tissue repair mechanisms, such as neurogenesis, angiogenesis and axonal growth, may be promising therapeutic approaches. Therapeutic agents can be delivered to the site of ischemic injury and many attempts have been made by intravenous injection, stereotactic and intraventricular administration [28,29]. In stroke patients, axons decrease in the peri-infarct area but begin to regrow at the end of the acute phase, returning to levels comparable to those of intact tissue during the chronic phase [30,31]. Therefore, the timely delivery of therapeutic agents during the acute phase is critical to reverse the molecular cascades that lead to neuronal demise. By contrast, therapies in the subacute and chronic phases should enhance regenerative processes, including axonal growth. In this study, we tested for the first time the neuroprotective effects of CHF6467, a painless recombinant NGF mutant, in a rat model of focal cerebral ischemia.

Various animal models of cerebral ischemia are available, each reproducing distinct aspects of the human condition. For instance, transient bilateral common carotid artery occlusion (tBCCAO) causes a diffuse reduction in cerebral blood flow to the forebrain, serving as a model of diffuse hypoperfusion injury and oxidative stress [32]; however, it does not induce a distinct, reproducibly measurable infarct. In contrast, tMCAo produces a focal, unilateral, territorial infarct characterized by a well-defined ischemic core and penumbra; this model replicates the vascular topography and lesion pattern of most human ischemic strokes, while also allowing for the highly reproducible measurement of infarct volume and lateralized sensorimotor deficits [33,34]. Since the present study aimed to quantify infarct volume and correlate it with neurological and lateralized sensorimotor outcomes, the tMCAo model was considered the most appropriate choice; nevertheless, tBCCAO remains a valuable tool for other purposes, such as studying global hypoperfusion and mechanisms associated with oxidative stress.

The results of the present study show that two intranasal administrations of CHF6467, immediately after the insult and 24 h later, improve motor and sensorimotor neurological deficits in an experimental model of focal cerebral ischemia in rats, suggesting improved motor recovery both at an early stage (i.e., after 48 h from the insult) and after seven days of pMCAo.

Motor and sensory improvements were associated with a significant reduction in infarct volume, confirming previous observations where neurological score has been shown to correlate with volume of hemispheric infarction from 24 h to 42 days after induction of ischemia [25,35,36,37].

The significant correlations between infarct volume, neurological scores, and sensorimotor performance (Figure S1 in Supplementary Materials) indicate that the behavioral improvements observed are directly associated with the extent of neuroprotection. Notably, the treatment effect was most pronounced at 24 h, when, for the same infarct volume, animals treated with CHF6467 exhibited significantly shorter adhesive removal times compared with the control group. At 48 h, the difference persisted, although there was greater overlap between the groups, likely due to spontaneous recovery. Data from the adhesive removal test were available for 16 animals per group because some rats were unable to complete the training procedure; however, this number remained consistent with the sample size estimated by the a priori power calculation. Importantly, exclusions were based exclusively on prespecified technical criteria related to task performance and were unrelated to treatment allocation. Rats subjected to pMCAO and treated with CHF6467 showed a 1.70-fold higher probability of achieving favorable functional recovery compared with the vehicle-treated animals. However, the reduction in sample size due to animal mortality (*n* = 3 in the vehicle-treated animals and *n* = 2 in the CHF6467-treated group) may have limited the statistical power and precision of the behavioral analyses, given that the sample size calculation called for a minimum of 16 animals per group.

Following intranasal administration, CHF6467 was detected in four out of five treated animals, with marked regional heterogeneity and the highest concentrations observed in the olfactory bulbs.

These results indicate that CHF6467 can reach the brain after intranasal administration; however, they do not confirm its concentration in the region most relevant to the behavioral outcomes assessed, nor do they provide evidence of its actual target engagement. Motor performance in the tests used (adhesive removal test, Bederson score) is primarily governed by cortical and hippocampal circuits; yet cortical CHF6467 was detected in only one animal, and hippocampal levels remained below the detection limit in all samples. Because tissue levels were measured at a single time point (2 h after the last dose), we cannot determine the temporal relationship between regional brain exposure and the neuroprotective effects observed at 48 h. This apparent discrepancy between limited drug detectability in the cortex and hippocampus at 2 h and the functional recovery observed in the tMCAo model may reflect: (i) local tissue exposure below the analytical detection threshold at 2 h that is nonetheless function-ally relevant; (ii) a temporal mismatch between the single sampling time point and the pharmacodynamically active exposure window; or (iii) a contribution of indirect or systemic mechanisms alongside, or in place of, direct engagement of cortical and hippocampal targets.

Nevertheless, previous studies indicate that CHF6467 reaches the hippocampus and cortex at later time points (6–24 h post-administration) [8], whereas accumulation in olfactory bulbs occurs within 30 min, consistent with the signals observed in this region. CHF6467 effectively reduced infarct volume and neurological deficits, but the present study did not aim to explore the molecular or cellular pathways involved (e.g., inflammatory cytokines, markers of microglial activation, neuronal apoptosis, oxidative stress, angiogenesis, or TrkA signaling) and therefore cannot establish the mechanism responsible for these effects.

Based on evidence from other experimental settings, we hypothesize that CHF6467 may activate anti-inflammatory and neuroprotective pathways [8,18,38]. Accordingly, Landucci et al. showed that intranasal administration of CHF6467 exerts its neuroprotective effects by reducing the elevation of cerebral mRNA levels of neuroinflammatory markers after hypoxic-ischemic encephalopathy [21]. Specifically, CHF6467 administered 5 min after the onset of oxygen-glucose deprivation (0.03 μg/mL) prevented neuronal death, restored synaptic transmission, and attenuated early excitotoxic and apoptotic events in vitro. Likewise, intranasal administration (20 μg/kg) 10 min after the hypoxic-ischemic insult was effective in neonatal models, supporting the hypothesis that early modulation of neuroinflammatory pathways may contribute to its neuroprotective effects [21].

Our data are consistent with previous studies using wild-type NGF. For instance, Zhu et al. found that intranasal administration of 0.6 mg/kg wild-type NGF, 24 h after cerebral reperfusion and continued for six consecutive days, induced neurogenesis in the ischemic striatum and functional recovery but did not reduce infarct volume at advanced stages [11]. By contrast, Li et al. demonstrated that the same dose of intranasal NGF for six consecutive days improved neurological function and reduced infarct volume only at day seven post-tMCAo [10].

Following a single intranasal administration of NGF, a reduction in infarct volume was only associated with an improvement in vestibular function but not in neurological deficit, at 24 and 48 h after the onset of tMCAo. In contrast, in our study, two CHF6467 administrations (immediately after injury induction and at 24 h after tMCAo) were able to reduce both neurological status and infarct volume.

### 4.1. Limitations of the Study

Our study demonstrates the neuroprotective efficacy of intranasal CHF6467 against ischemic brain injury, as evidenced by both functional recovery and a significant reduction in infarct volume. However, several limitations must be considered when interpreting these findings:

(1) Pharmacokinetic and regional exposure variability: Although CHF6467 was detected in the brains of four out of five treated animals following intranasal administration, its distribution across regions was heterogeneous at the single evaluated time-point (2 h post-dose) in a limited cohort. While these data confirm that the drug reaches the central nervous system, the marked inter-individual variability and absence of a complete pharmacokinetic profile prevent establishing a direct, quantitative relationship between regional brain exposure and the functional and histological neuroprotection observed at 48 h; these two sets of results should therefore be considered complementary rather than mechanistically linked.

(2) Sample size constraints in behavioral assessments: Not all animals completed the entire battery of behavioral tests, which may have reduced the precision of these secondary endpoints. In the acute tMCAo cohort, *n* = 16 animals per group completed the adhesive removal test (out of an initial *n* = 20 per group). In the long-term pMCAo cohort, neurological evaluation was completed in 13 rats in the vehicle group and 14 in the CHF6467 group. Falling below the pre-established threshold of 16 animals per group due to peri-operative mortality, this sub-analysis may have been underpowered to detect smaller treatment effects.

(3) Acute design vs. long-term repair mechanisms: A fundamental conceptual limitation is that the acute study focused on a 48-h endpoint, capturing acute neuro-protection (prevention of cell death and early functional deficits) rather than long-term tissue repair or regenerative processes (such as neuroplasticity, angiogenesis, or neuro-genesis).

(4) Lack of molecular and cellular endpoints: Our proposed anti-inflammatory and cellular mechanisms remain speculative, as no direct molecular or cellular markers (e.g., cytokine profiling, microglial activation markers, or neurogenic indicators) were measured in this specific cohort.

(5) Translational scope: Finally, the study was conducted exclusively on young adult male rats using a single dose and dosing regimen. Given that stroke predominantly affects older populations and exhibits distinct sex-dependent pathophysiology, these results cannot be directly generalized to aged or female cohorts.

### 4.2. Conclusions and Future Directions

Further evaluations are needed to define the practical and translational relevance of CHF6467 in ischemic stroke. This includes examining pharmacokinetic sampling at multiple time points, assessing molecular and histological mechanistic endpoints, conducting prolonged functional and histological follow-up (7–28 days), and considering factors such as sex and dosage.

## 5. Patents

A patent related to this work has been filed (Patent No. WO 2026/022244 A1).

## Figures and Tables

**Table 1:** **Regional brain biodistribution of CHF6467 two hours post-intranasal administration.** CHF6467 concentration, expressed as pg/mg of protein (mean ± SD), in olfactory bulbs, cortex, striatum and hippocampus from intact rats treated twice with CHF6467 or vehicle. Samples were collected 2 h after the second drug administration. Measurements were performed in duplicate and repeated at least twice.

Rat ID	Treatment Group	Olfactory Bulb	Cortex	Striatum	Hippocampus
1	CHF6467	>25 (out of scale)	0.72 ± 0.02	n.d.	n.d.
4	CHF6467	n.d.	n.d.	n.d.	n.d.
6	CHF6467	n.d.	n.d.	3.25 ± 1.43	n.d.
8	CHF6467	18.62 ± 0.88	n.d.	n.d.	n.d.
10	CHF6467	5.53 ± 1.12	n.d.	n.d.	n.d.
3	vehicle	n.d.	n.d.	n.d.	n.d.
5	vehicle	n.d.	n.d.	n.d.	n.d.
7	vehicle	n.d.	n.d.	n.d.	n.d.
9	vehicle	n.d.	n.d.	n.d.	n.d.
Detectability (CHF6467) Proportion (n/N)		3/5 (60%)	1/5 (20%)	1/5 (20%)	0/5 (0%)
Mean ± SD (CHF6467) Quantifiable rats		12.08 ± 9.26 *	n.a. (*n* = 1)	n.a. (*n* = 1)	n.a. (*n* = 0)

Data are presented as individual values (Mean ± SD of analytical replicates) or group summaries. n.d. = not detectable (below lower limit of detection); n.a. = not applicable. * Mean ± SD for the olfactory bulb is calculated from quantifiable samples (Rats 8 and 10). If Rat 1 (>25 ng/g) is included using the conservative lower bound of 25 ng/g, the overall mean ± SD for positive olfactory bulb samples is 16.38 ± 9.87 ng/g.

## Data Availability

All data associated with this study are present in the paper.

## Supplementary Materials

The following supporting information can be downloaded at https://www.mdpi.com/article/10.3390/brainsci16080839/s1, Figure S1: Correlation analysis. Infarct volume is positively correlated with neurological score (Number of XY pairs = 40) (a), time in contralateral adhesive tape removal (Number of XY pairs = 32) at 24 h (b) and 48 h (c). Neurological score is positively correlated with time in contralateral adhesive tape removal (Number of XY pairs = 32) at 24 h (d) and 48 h (e).

## Abbreviations

The following abbreviations are used in this manuscript:

ART	adhesive removal test
BBB	blood-brain barrier
BBS	baseline behavioral score
NGF	Nerve growth factor
S/N	Signal-to-noise ratio
pMCAo	permanent middle cerebral artery occlusion
tBCCAO	transient bilateral common carotid artery occlusion
tMCAo	transient middle cerebral artery occlusion
TTC	2,3,5-triphenyltetrazolium chloride
