# Supplementary material for: CHF6467 Exerts Neuroprotective Effects in the Rat Model of Acute Ischemic Stroke
Source: Brain Sci. 2026 Aug 7;16(8):839. doi: 10.3390/brainsci16080839 (PMC13510487; doi:10.3390/brainsci16080839)
Supplement: Supplementary file 1 [file brainsci-16-00839-s001.zip › brainsci-4431216-supplementary.pdf]

Brain Sciences

SUPPLEMENTARY MATERIALS

CHF6467 shows neuroprotective effects in the rat model of acute ischemic stroke

Chiara Demartini [1], Fabrizio Facchinetti [2], Fabio Blandini [3,4], Cristina Tassorelli [1,3], Francesca Malerba [5,6], Antonino Cattaneo [5], Diana Amantea [7], Bruno P. Imbimbo [2] and Rosaria Greco [1,*]

[1] Section of Translational Neurovascular Research, IRCCS Mondino Foundation; via Mondino, 2, 27100 Pavia, Italy; chiara.demartini@mondino.it; rosaria.greco@mondino.it

[2] Research & Development, Chiesi Farmaceutici; Largo Belloli 11/A, 43122 Parma, Italy; F.Facchinetti@chiesi.com; B.Imbimbo@chiesi.com

[3] Department of Brain and Behavioral Sciences, University of Pavia; via Bassi 21, 27100 Pavia, Italy; cristina.tassorelli@unipv.it

[4] Ca'Granda IRCCS Foundation-Ospedale Maggiore Policlinico; Via Francesco Sforza 28, 20122 Milan, Italy; fabio.blandini@policlinico.mi.it

[5] Fondazione EBRI - Rita Levi-Montalcini; Viale Regina Elena, 295, 00161 ROMA, Italy; f.malerba@ebri.it; a.cattaneo@ebri.it;

[6] Institute of Nanotechnology National Research Council (CNR Nanotec) c/o Campus Ecotekne; via Monteroni, 73100 Lecce, Italy

[7] Section of Preclinical and Translational Pharmacology, Department of Pharmacy, Health and Nutritional Sciences, University of Calabria, via Savinio, I-87036 Rende, CS, Italy.

* Correspondence: rosaria.greco@mondino.it; Tel.: 0039-(0)382-380255

Correlation analysis

The correlation analysis showed that infarct volume is directly correlated with neurological deficit. Specifically, a higher infarct volume is associated with higher neurological score (Figure S1a) and more time in contralateral adhesive tape removal (at both 24 and 48h) (Figure S1b and c). Of interest, the two behavioral tests are also positively correlated (Figure S1d and e).

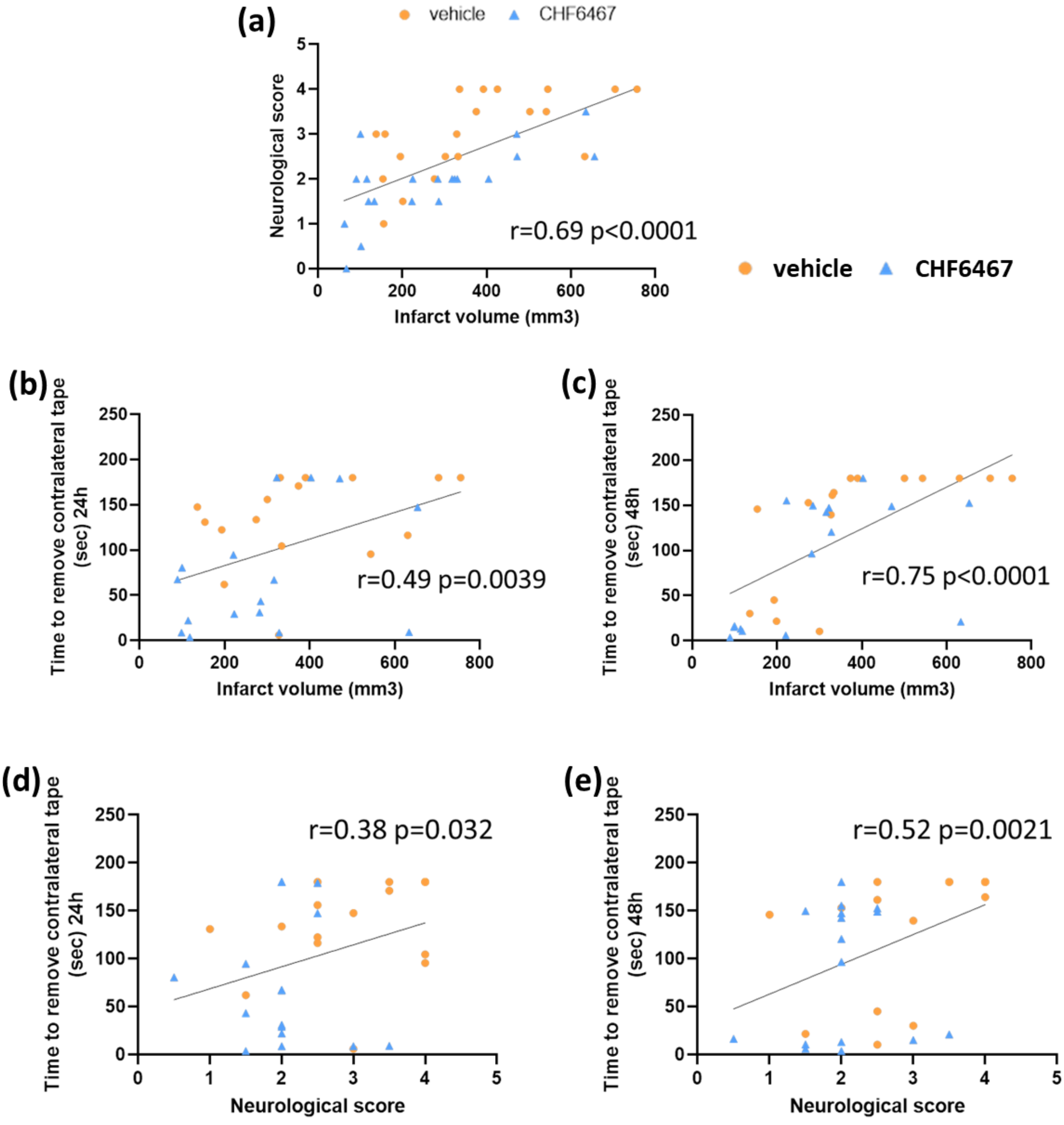

Figure S1. Correlation analysis. Infarct volume is positively correlated with neurological score (Number of XY pairs=40) (a), time in contralateral adhesive tape removal (Number of XY pairs=32) at 24h (b) and 48h (c). Neurological score is positively correlated with time in contralateral adhesive tape removal (Number of XY pairs=32) at 24h (d) and 48h (e).
